# A proxy measure of clinical insight in psychosis: an electronic health records-based validation study

**DOI:** 10.1192/bjo.2025.10850

**Published:** 2025-10-01

**Authors:** Álvaro López-Díaz, Javier-David Lopez-Morinigo, Víctor Ortiz-García de la Foz, Helena Marín-Mateo, Maria Dolores Ortíz-Jiménez, Jeff David Huarcaya-Victoria, Gerardo Gutiérrez-Talavera, César González-Blanch, Benedicto Crespo-Facorro, Manuel Jesús Cuesta, Rosa Ayesa-Arriola

**Affiliations:** Mental Health Clinical Management Unit, University Hospital Virgen Macarena, Spain; Translational Psychiatry Research Group (PsyNal), Seville Biomedical Research Centre (IBiS), Spanish Network for Research in Mental Health, Carlos III Institute (CIBERSAM ISCIII), Department of Psychiatry, School of Medicine, University of Seville, Spain; Department of Child and Adolescent Psychiatry, Institute of Psychiatry and Mental Health, General University Hospital Gregorio Marañón (IiSGM), Spanish Network for Research in Mental Health, Carlos III Institute (CIBERSAM ISCIII), School of Medicine, Complutense University of Madrid, Spain; Southeast University Hospital, Arganda del Rey, Madrid, Spain; Department of Psychiatry, University Hospital Marqués de Valdecilla, Valdecilla Health Research Institute (IDIVAL), Spanish Network for Research in Mental Health, Carlos III Institute (CIBERSAM ISCIII), School of Medicine, University of Cantabria, Santander, Spain; Professional School of Human Medicine, San Juan Bautista Private University, Ica, Perú; Department of Psychiatry, University Hospital of Navarra, Pamplona, Spain; Department of Psychology, International University of La Rioja (UNIR), Logroño, Spain; Mental Health Clinical Management Unit, University Hospital Virgen del Rocío, Seville, Spain; Navarra Institute of Health Research (IdiSNA), Pamplona, Spain

**Keywords:** Insight assessment, electronic health records, proxy measures, psychosis

## Abstract

**Background:**

Insight assessment in psychosis remains challenging in practice-oriented research.

**Aims:**

To develop and validate a proxy measure for insight based on information from electronic health records (EHR). For that purpose, we used data on the Scale to Assess Unawareness of Mental Disorder (SUMD) and data from EHR notes of patients in an early psychosis intervention programme (Programa de Atención a Fases Iniciales de Psicosis, Santander, Spain).

**Method:**

Junior and senior clinicians examined 134 clinical notes from 106 patients to explore criterion and content validity between SUMD and a clinician-rated proxy measure, using three SUMD items.

**Results:**

In terms of criterion validity, SUMD scores correlated with the proxy (*r* = 0.61, *P* < 0.001), even after adjusting for the following confounders: type of psychotic disorder, clinical remission status and rater experience (*r* = 0.58, *P* < 0.001); and the proxy predicted good insight status (odds ratio 20.95, 95% CI 7.32–59.91, *P* < 0.001). Regarding content validity, the three main SUMD subscores correlated with the proxy (*r* = 0.55–0.60, *P* < 0.005). There were no significant differences in age, gender or other clinical variables, i.e. discriminant validity, and the proxy significantly correlated with validated psychometric instruments, i.e. external validity. Intraclass correlation coefficient (i.e. interrater reliability) was 0.88 (95% CI 0.59–1.00, *P* < 0.05).

**Conclusions:**

This SUMD-based proxy measure was shown to have good to excellent validity and reliability, which may offer a reliable and efficient alternative for assessing insight in real-world clinical practice, EHR-based research and management. Future studies should explore its applicability across different healthcare contexts and its potential for automation, using natural language-processing techniques.

A multicentre study conducted by the World Health Organization in 1973 demonstrated that the majority of individuals diagnosed with schizophrenia exhibited a marked lack of insight,^1^ which was subsequently replicated.^2^ Impaired insight has thus become a core feature of psychosis from early phases and is associated with premorbid personality traits.^3^ The multidimensional models of insight – illness awareness, symptom relabelling, treatment compliance and awareness of the social consequences, proposed in the early 1990s^7,8^ – led to three decades of extensive research in the area.^9^ Thanks to this work, insight in psychosis has been consistently linked to positive outcomes – greater insight and better outcomes, namely reduced psychotic symptom severity,^10^ reduced use of coercive treatments^9^ and better psychosocial functioning.^11,12^ More controversially, insight gain may result in depression,^10,13^ increased risk of suicidal behaviour^14–17^ and poorer self-perceived quality of life.^18^ Although so-called insight paradox^19^ remains far from clear,^20^ the aforementioned strong association of insight with positive outcomes results in insight assessment becoming a matter of major clinical relevance.^21^

Insight measurement can be challenging in both routine clinical practice and research, especially given its conceptual complexity. The first insight assessments relied on patients’ narrative accounts of their mental health issues, which could not be used for research purposes. To address this issue, investigators designed interviews that classified patients as having ‘good’ or ‘poor’ insight: for instance, the Present State Examination.^22^ Subsequently, semi-structured scored interviews were validated to measure insight unidimensional, such as the Insight and Treatment Attitudes Questionnaire (ITAQ).^23^ Finally, in order to capture the aforementioned multidimensionality of insight,^7,8^ two scales were validated, namely the Scale to Assess Unawareness of Mental Disorder (SUMD)^24^ and its more commonly used shortened version,^2^ and the Schedule for Assessment of Insight (SAI),^8^ which also had an expanded version (SAI-E).^25^ Further insight assessments include self-reports such as the Birchwood Insight Scale,^26^ the Markova and Berrios Insight Scale (MBIS)^27^ and the specific insight item of two large scales of general psychopathology, the Positive and Negative Symptoms Scale (PANSS)^28^ and the Manual for the Assessment and Documentation of Psychopathology (AMDP system).^29^ However, these insight assessments require considerable time input and must be rated by trained staff.^30^ Moreover, some degree of cooperation is required that may introduce a selection bias into research studies, namely the exclusion of potentially eligible participants with poor insight, especially in randomised controlled trials.^31^

These limitations of previous approaches to insight measurement are more pronounced in real-world epidemiological studies, given the scant incorporation of psychometric tests into daily clinical practice.^30^ Alternatively, insight in psychosis (hereafter referred to as simply insight) could be assessed in time-constrained clinical settings with proxy measures, which can be defined as ‘indirect measures of the outcome of interest (i.e. insight), with which there is strong correlation’. Proxy measures are commonly used when the outcome variable cannot be directly observed/assessed,^32^ and have had their utility proven in mental health science research. A variety of proxy measures have been developed and validated;^33,34^ those derived from either sociodemographic variables or unstructured electronic health records (EHR) data have proved particularly useful in practice-oriented research studies.^35,36^ Nevertheless, the widespread implementation of proxy measures in routine clinical care and EHR-based research remains a pending subject.

## Aims

The objective of this study was to assess the validity and reliability of a SUMD-based proxy measure for insight assessment in psychosis using EHR. Specifically, we examined the extent to which a three-level clinician rating of insight, derived from routine EHR notes, aligns with SUMD scores in subjects with psychosis. If validated, this approach could provide a valuable tool for both routine clinical practice and real-world EHR-based research.

## Method

### Study population and inclusion/exclusion criteria

Data for this study were retrieved from the 3-year follow-up, first-episode psychosis (FEP) Programa de Atención a Fases Iniciales de Psicosis (PAFIP) cohort, a publicly funded early intervention service (EIS) for people with psychosis, at Marqués de Valdecilla University Hospital (Santander, Spain). A detailed description of PAFIP is available elsewhere.^37,38^ Briefly, referrals to PAFIP came from local/regional in- and out-patient mental health services, emergency departments and primary care trusts in Cantabria (on the northern coast of Spain, with a catchment area population estimated at approximately 535 000). Potentially eligible candidates were screened against the following inclusion criteria: (a) age between 16 and 60 years, both inclusive; (b) living in the catchment area; (c) experiencing a FEP as outlined below; (d) meeting DSM-IV criteria for a non-affective psychotic disorder, which were confirmed by the Structured Clinical Interview for the DSM; and (e) no previous treatment with antipsychotic medication or, if previously treated, a total treatment duration of less than 6 weeks. Drug dependence (excluding nicotine), having an IQ <70 according to the Wechsler Adult Intelligent Scale-III vocabulary subtest and a history of neurological disease or head injury were exclusion criteria. The project was approved by the local research ethics committee (CEIm Cantabria; internal code of approval, no. 2014.245) in accordance with international standards for research ethics. Participants provided written informed consent as approved by the local research ethics committee. The characteristics of the PAFIP cohort were reported to be generalisable to the target population.^38^ This study was reported in accordance with the Strengthening the Reporting of Observational Studies in Epidemiology (STROBE) Statement guidelines.^39^

### Selected demographic, clinical and psychometric variables

Data on demographic variables, premorbid characteristics and clinical status were collected from face-to-face interviews with patients, key informants and medical records at the time of PAFIP inception. Sociodemographic variables included age, gender, ethnicity, marital or cohabitation status, employment status, educational level, urbanicity status (classified as living in a municipality with more than 10 000 inhabitants), socioeconomic status and living arrangements at the onset of psychosis. Clinical characteristics included family history of psychosis, history of substance use (defined as harmful or hazardous use of alcohol, cannabis or other illicit drugs), premorbid adjustment, premorbid IQ, age at onset of psychosis, duration of untreated psychosis (DUP), disease duration (from the onset of psychosis until clinical records were examined for SUMD proxy scoring), level of psychosocial functioning and neurocognitive performance and type of psychotic disorder (i.e. DSM-IV coding).

Socioeconomic status was assessed using the Hollingshead and Redlich scale. Premorbid psychosocial adjustment was measured using the Cannon–Spoor Premorbid Adjustment Scale (PAS), which, for the sake of interpretation, was dichotomised into good/poor premorbid adjustment through the median. The Wechsler Adult Intelligence Scale (third edition, WAIS-III) Vocabulary Subtest estimated premorbid IQ. Social functioning was assessed with the Disability Assessment Schedule (DAS). The level of global cognitive functioning (GCF) provided an overall measure of neurocognitive performance.

PAFIP full assessments were carried out at baseline, 6 weeks, 12 months and 36 months, in accordance with the PAFIP protocol. In addition, follow-up clinical appointments were arranged on a monthly basis over the follow-up. Psychopathological symptoms were assessed using the expanded version of the Brief Psychiatric Rating Scale (BPRS), the Scale for the Assessment of Positive Symptoms (SAPS) and the Scale for the Assessment of Negative Symptoms (SANS). Symptomatic remission was defined according to the criteria set out by Andreasen et al.^40^

### Insight assessment

Lack of insight was measured with the shortened version of SUMD. Specifically, the three general items of the shortened version of SUMD – (a) awareness of having a mental disorder, (b) awareness of the need for treatment and (c) awareness of the social consequences of the disorder – were considered. Scores for each item ranged from 1 to 5, with higher scores indicating poorer insight, which were summed to create total SUMD scores ranging from 3 to 15. Those with a SUMD total score of 6 or below had ‘good insight’, while a total score of 7 or above indicated ‘poor insight’.^41^ SUMD has been widely used in both clinics and research, and was demonstrated to have good reliability and validity.^41,42^

### Proxy measures for insight assessment from SUMD

**Fig. 1:**
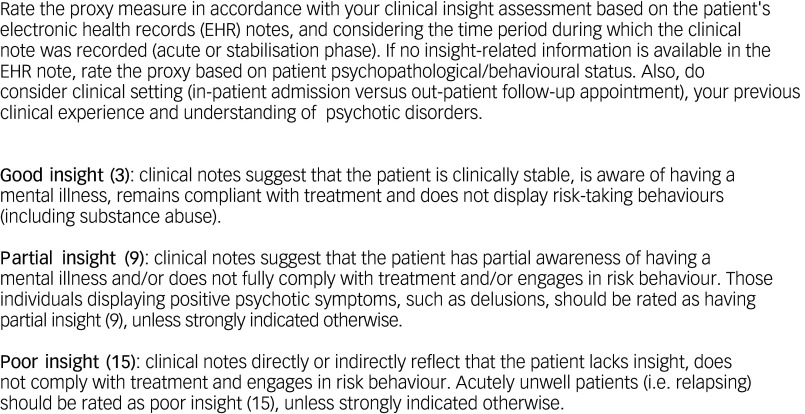
Scale to Assess Unawareness of Mental Disorder (SUMD) proxy-based assessment: examiner’s guide.

**Fig. 2:**
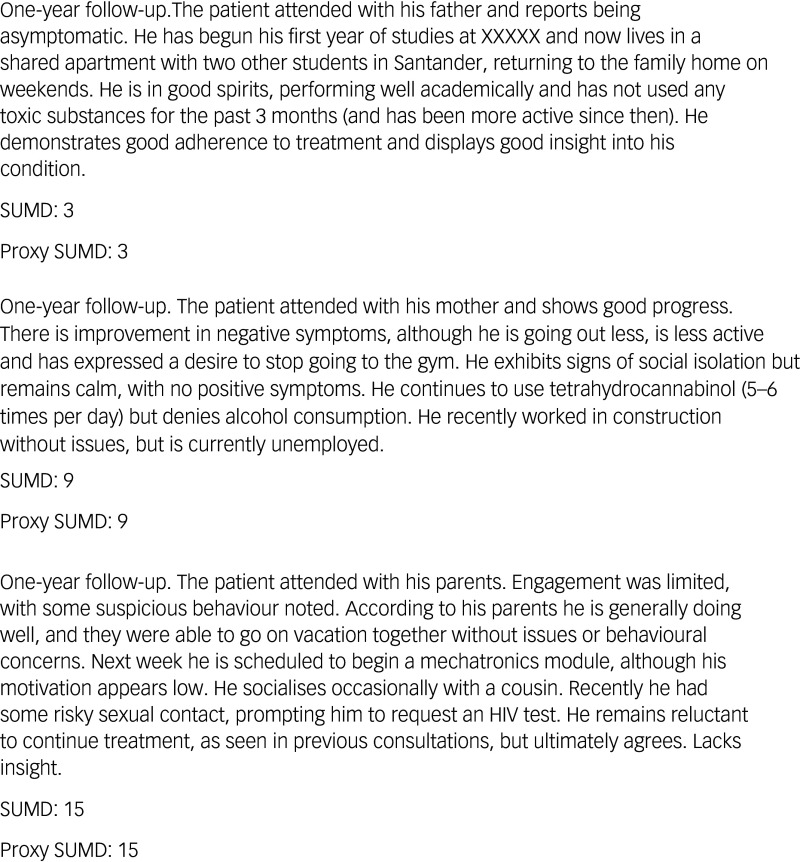
Examples of clinical notes and Scale to Assess Unawareness of Mental Disorder (SUMD) proxy scoring method.

The proxy version of SUMD is shown in Fig. 1, according to which, total insight scores were classified unidimensional on a 3-point Likert scale as either good (score of 3), partial (score of 9) or poor insight (score of 15). To rate the SUMD proxy, clinicians carefully read the patient’s EHR and considered their clinical status at that point (acutely psychotic or clinically stable). Where no insight assessment was documented (which is relatively common in routine clinical practice due to time constraints during visits), raters considered patient psychopathological/behavioural information, as well as clinical setting (in-/out-patient) and their clinical experience and understanding of psychotic disorders, to provide scores on the SUMD proxies. Some representative examples of the scoring method are shown in Fig. 2.

### Standardised procedure for validation of the SUMD proxy measure

For study purposes, the proxy version of SUMD was validated using EHR and psychometric assessments from the 1- and 3-year follow-up appointments of the PAFIP programme. Raters were trained in the use of the proxy tool, and followed a specific guide designed by the first author (L.-D.A.). Raters’ experience (less than or over 5 years) was recorded and tested as a potential moderator of proxy scoring. Cases were selected if: (a) EHR were available at the time of the 1- and 3-year follow-up PAFIP psychometric assessments; (b) the clinician recording the patient’s mental state (i.e. EHR) was not the evaluator who conducted the psychometric assessment; (c) the SUMD score was unavailable in the EHR; and (d) the third clinician evaluating the EHR to rate the proxy measure of insight was blinded to the SUMD scores. If an EHR lacked sufficient information pertaining to the proxy insight assessment (e.g. ‘no changes from previous visit’), the closest valid EHR (within a 1-month time frame) to the SUMD assessment was used instead. In order to evaluate the psychometric validity of the proxy measures, the degree of concordance between SUMD and proxy scores from EHR was examined using a variety of statistical methods detailed below.

### Data analysis

Descriptive statistics were used to examine the demographic and clinical characteristics of the sample, which are reported as either percentages, mean or median and standard deviation or interquartile range (IQR), as appropriate. Test statistics of the proxy measure were specifically assessed using four types of psychometric validity: criterion, content, discriminant and external validity, and interrater reliability.

In order to test the criterion validity (i.e. the extent to which the proxy measure was correlated with SUMD scores), both the concurrent and predictive validity were examined. First, unadjusted univariate correlation analyses between proxy SUMD and SUMD scores were performed and reported as Pearson’s or Spearman’s coefficient, depending on whether the variable followed a normal distribution, respectively, according to the Kolmogorov–Smirnov normality test. Second, partial correlations were run between proxy SUMD and SUMD scores while controlling for potential confounders, such as type of psychotic disorder (first-episode schizophrenia versus all other psychotic disorder diagnoses), clinical status (remission versus non-remission status according to Andreasen’s criteria) and the rater’s previous experience in scoring proxies (less than or more than 5 years). Finally, the extent to which the proxy SUMD predicted those cases with good/poor insight was determined by a binary logistic regression analysis (using the above cut-off points). Odds ratios and the corresponding 95% confidence intervals were estimated. Nagelkerke’s *R*^2^ was used to measure the goodness of fit of the resulting model, and the area under the receiver operating characteristic (ROC) curve (AUC) evaluated the classifier’s performance of the proxy.

For content validity (i.e. the extent to which the proxy assessed and represented all facets of the abbreviated SUMD construct), the correlation coefficients between proxy SUMD scores and those for the three main insight domains of the SUMD were examined. Discriminant validity (i.e. the extent to which the proxy measure was not related to other clinical variables or different constructs) was determined by comparing proxy SUMD median scores across patients’ age, gender, educational level, living status and illness duration. External validity (i.e. the extent to which the results of the proxy tool can be generalised or transferred to other psychometric tests) was assessed by bivariate correlations between proxy SUMD scores and BPRS, SAPS, SANS and DAS scores.

The interrater reliability of the proxy method was evaluated using the intraclass correlation coefficient (ICC), a two-way random-effects model and absolute agreement, which was calculated from three clinical notes randomly selected and rated by six researchers from all participating centres.

The handling of missing data was conducted in accordance with the pairwise deletion method. The level of significance was set at *P* < 0.05. Post hoc power analyses were performed using G-Power software (version 3.1.9.4, Franz Faul, University of Kiel, Germany), assuming a medium effect size (*P* = 0.3) and *α* = 0.05. The remaining statistical analyses were conducted with IBM SPSS Statistics software, version 29 for MacOS.

## Results

### Sample characteristics

A total of *N* = 134 clinical notes from *n* = 106 patients from the PAFIP programme were included. The majority of the clinical notes (57.5%, *n* = 77) were from the PAFIP 1-year follow-up appointment, 46.3% (*n* = 62) were from first-episode schizophrenia cases and 59% (*n* = 79) were from patients in remission. Seventy-six (56.7%) EHR notes were assessed by experienced clinicians (i.e. with >5 years of clinical experience) for proxy insight scoring. Sociodemographic and clinical features of the cases analysed are shown in Table 1.

**Table 1:** Sociodemographic, clinical and psychometric characteristics of the cases analysed

Characteristics	Total sample (*N* = 134)^a^
**Sociodemographics**	
Age (years), median (IQR)	34.57 (23.24–42.60)
Gender (male), *n* (%)	71 (53)
Ethnicity (European White), *n* (%)	117 (87.3)
Marital status (unmarried and without a partner), *n* (%)	87 (64.9)
Employment status (unemployed and not studying), *n* (%)	42 (31.3)
Education (secondary or lower education), *n* (%)	110 (82.1)
Socioeconomic status of parents (low), *n* (%)	76 (56.7)
Urban area, *n* (%)	96 (71.6)
Living arrangements (alone), *n* (%)	14 (10.4)
**Clinical features**	
Family history of psychosis, *n* (%)	34 (25.4)
Substance use (yes), *n* (%)	76 (56.7)
Premorbid adjustment (poor)^b^, *n* (%)	68 (50.7)
Premorbid IQ, median (IQR)	100 (90.00–106.25)
Age at onset (years), median (IQR)	32.34 (22.83–41.81)
Duration of untreated psychosis (months)	3 (0.50–10.00)
Disease duration (years), median (IQR)	3 (1.12–3.3)
Level of social functioning (DAS), median (IQR)	0 (0–2)
Global Cognitive Functioning, median (IQR)	0.64 (0.18–1.25)
Type of psychotic disorder (first-episode schizophrenia), *n* (%)	62 (46.3)
**Psychotic symptoms characteristics**	
Brief Psychiatric Rating Scale (BPRS) total score, median (IQR)	26 (24–33)
Scale for the Assessment of Positive Symptoms (SAPS) total score, median (IQR)	0 (0–0)
Scale for the Assessment of Negative Symptoms (SANS) total score, median (IQR)	2 (0–8)
Symptomatic remission (Andreasen criteria), *n* (%)	79 (59)
Scale to Assess Unawareness of Mental Disorder (SUMD), median (IQR)	3 (3–7)

DAS, Disability Assessment Schedule; IQR, interquartile range.

a. A total of 134 clinical notes from 106 patients were included in the analyses.

b. The median score (2.05) on the Premorbid Adjustment Scale (PAS) was used as the cut-off point for determination of good premorbid adjustment.

The psychometric characteristics of the sample are summarised in Table 1. The median SUMD total score was 3 (IQR 3–7) and the median SUMD proxy score was 3 (IQR 3–9). In terms of good insight categorisation, 71.6% (*n* = 96) of patients assessed with SUMD were classified as having good insight compared with 58.2% (*n* = 78) who met the proxy criteria. The post hoc power analysis (1 – *b* error probability) yielded a value of 0.95, indicating that the sample size had sufficient statistical power to detect significant associations between SUMD and its proxy version.

### Criterion validity

SUMD score significantly correlated with its proxy (*r* = 0.61, 95% CI 0.48–0.71, *P* < 0.001). After controlling for confounders, the partial rank correlation coefficient result was similar (*r* = 0.58, 95% CI 0.45–0.68, *P* < 0.001), thus indicating good concurrent validity of the proxy tool. Regression analysis showed that the proxy SUMD predicted good insight status (odds ratio 20.95, 95% CI 7.32–59.91, *P* < 0.001), with the model accounting for 42% of the variance on insight status. In total, 79.1% of cases were correctly classified in terms of good/poor insight. The AUC was 0.76 (95% CI 0.67–0.85, *P* < 0.001), indicating that the proxy SUMD had accurate predictive validity.

### Content and discriminant and external validity

Correlation coefficients between the SUMD proxy and each of the three SUMD domains (awareness of mental disorder, awareness of need for treatment and awareness of the social consequences) ranged from *r* = 0.55 to *r* = 0.60 (all of which were significant at *P* < 0.001), indicating good content validity. No significant differences in age, gender, educational level, living arrangement or disease duration were observed, demonstrating excellent discriminant validity of the proxy version. SUMD proxy scores showed low to medium significant correlations with BPRS (*r* = 0.33, 95% CI 0.17–0.48, *P* < 0.001), SAPS (*r* = 0.49, 95% CI 0.34–0.61, *P* < 0.001), SANS (*r* = 0.17, 95% CI 0–0.34, *P* < 0.047) and DAS scores (*r* = 0.21, 95% CI 0.03–0.37, *P* < 0.018), indicating optimal external validity of the proxy tool.

### Interrater reliability

The ICC of the SUMD proxy was 0.88 (95% CI 0.59–1.00, *P* < 0.001), which indicated good interrater reliability. The psychometric validity results are detailed in Table 2.

**Table 2:** Summary of SUMD scores and psychometric properties of its proxy version

Summaries	Scores
**Summary of SUMD assessments**	
G1: insight into having a mental disorder, median (IQR)	3 (1–3)
G2: insight into the need for treatment, median (IQR)	3 (1–2)
G3: insight into the social consequences of the disorder, median (IQR)	3 (1–2)
SUMD total score (G1 + G2 + G3), median (IQR)	3 (3–7)
Percentage of cases categorised as ‘good insight’, *n* (%)	96 (71.6)
**Summary of proxy SUMD assessments**	
Proxy SUMD scores, median (IQR)	3 (3–9)
Percentage of cases categorised as ‘good insight’, *n* (%)	78 (58.2)
**Summary of the psychometric validity of proxy SUMD**	
* Criterion validity*	
Spearman correlation coefficient with SUMD total score, rho (95% CI)	0.61 (0.48–0.71)*
Partial rank correlation coefficient with SUMD total score (95% CI)	0.58 (0.45–0.68)*
Predictive ability for SUMD ‘good insight’ classification, OR (95% CI)	20.95 (7.32–59.91)*
Nagelkerke *R*^2^ of the prediction model	0.42
Overall percentage of SUMD ‘good insight’ correct predictions (%)	79.1
AUC of the model (95% CI)	0.76 (0.67–0.85)*
* Content validity*	
Insight into having a mental disorder, rho (95% CI)	0.60 (0.47–0.70)*
Insight into the need for treatment, rho (95% CI)	0.55 (0.42–0.66)*
Insight into social consequences of the disorder, rho (95% CI)	0.58 (0.46–0.69)*
* Discriminant validity*	
Age, rho (95% CI)	0.28 (−0.15 to 0.20)^NS^
Gender, RPB (95% CI)	−0.01 (−0.18 to 0.17)^NS^
Educational level, RPB (95% CI)	0.12 (−0.05 to 0.29)^NS^
Living arrangement, RPB (95% CI)	0.03 (−0.14 to 0.21)^NS^
Disease duration, rho (95% CI)	−0.26 (−0.21 to 0.16)^NS^
* External validity*	
Brief Psychiatric Rating Scale (BPRS), rho (95% CI)	0.33 (0.17–0.48)*
Scale for the Assessment of Positive Symptoms (SAPS), rho (95% CI)	0.49 (0.34–0.61)*
Scale for the Assessment of Negative Symptoms (SANS), rho (95% CI)	0.17 (0–0.34)*
Disability Assessment Schedule (DAS), rho (95% CI)	0.21 (0.03–0.37)*
* Interrater reliability*	
Intraclass correlation coefficient, ICC (95% CI)	0.88 (0.59–1.00)

AUC, area under the curve; OR, odds ratio; IQR, interquartile range; NS, not significant; RPB, point biserial correlation; SUMD, Scale to Assess Unawareness of Mental Disorder.

*P < 0.05.

## Discussion

### Main findings

This study aimed to assess the validity and reliability of a SUMD-based proxy measure for insight assessment in psychosis using EHR. To this end, EHR and psychometric assessments from a 3-year follow-up FEP cohort of patients under an EIS (PAFIP programme) from Santander (Spain) were used. In order to evaluate the validity of this proxy tool, the concordance between SUMD and its proxy version scores was examined while controlling for the effects of other explanatory variables, including the type of psychotic disorder (first-episode schizophrenia versus all other diagnoses), in-/out-patient status and raters’ experience. Of relevance, the SUMD-based proxy measure was demonstrated to have robust construct and content validity with SUMD, along with excellent discriminant validity against other unrelated constructs and optimal external validity in relation to other validated instruments for assessment of symptom severity and disability. To the best of our knowledge, this is the first study to validate an EHR-based proxy tool for the assessment of insight in patients with psychotic disorders under real-world conditions.

Overall performance of this insight proxy measure, including four types of validity – criterion, content, discriminant and external validity, and interrater reliability – was found to be good to excellent. In terms of construct validity, the psychometric properties of the SUMD proxy fulfilled quality standards for the validation of proxy tools.^32^ When compared with other validated clinician-rated proxy measures in the field of psychosis, the correlation coefficient for the proxy SUMD (*r* = 0.61) was slightly higher than in a previous validation study of a proxy measure of PAS (*r* = 0.57).^35^ Of note, this proxy version of SUMD correctly classified more than 3 of 4 individuals in terms of good/poor insight status (79.1%), in line with external validation of the PANSS-6, Young Mania Rating Scale 6 (YMRS-6) and Montgomery–Asberg Depression Rating Scale 6 (MADRS-6) proxies, which ranged from 76.7 to 83.3%.^36^ The AUC for the proxy SUMD (0.76) demonstrated slightly enhanced discriminative power compared with a proxy measure of the PANSS remission criteria based on Clinical Global Impressions Scale (CGI) scores (0.73).^43^ In terms of interrater reliability, the SUMD proxy method ICC (0.88) was higher than that in a previous validation study of proxy versions of PANSS-6, YMRS-6 and MADRS-6.^36^ Finally, outside the framework of clinician-rated proxy tools but within the realm of third-party proxy tools, it should be noted that the EHR-based proxy SUMD had a stronger correlation (*r* = 0.61) with SUMD than the validated caregivers’ version of this scale (*r*^i^ = 0.48).^44^ Above and beyond significant differences between clinician- and caregiver-rated scales,^45^ this finding suggests that the proxy SUMD version may provide a more accurate estimate of patient insight from review of EHR than the daily impressions of their close relatives or key informants. Taken together, these comparisons appear to indicate that the proxy SUMD may become a valid and reliable instrument for measurement of clinical insight in psychotic disorders, in both daily practice and EHR-based research.

### A novel contribution to insight assessment in psychosis

In 1934, Sir Aubrey Lewis characterised insight as ‘a correct attitude to a morbid change in oneself’, emphasising that it could be inferred only from the patient’s behaviour, particularly their verbal expressions.^46^ Based on the multidimensional models of insight proposed in the 1990s,^7,8^ scales measuring multiple insight dimensions, such as SUMD and the SAI-E, were validated.^24,25^ In spite of the conceptual complexity involved, these validation studies demonstrated that insight can be measured, especially given the good level of agreement between these scales.^21,47^ In today’s mental health crisis, however, clinicians could be tempted to avoid time-related issues, hence assessing insight in an unidimensional manner as present/absent, which cannot generate data for research purposes and may prevent them from monitoring potential insight changes over time. By building on previous validation studies of EHR-based proxy measures in psychosis,^35,36^ the present study developed and validated a SUMD-based proxy tool for the assessment of insight in psychosis, which provides an overall measure of patients’ insight level from EHR. This clinical insight information could be considered a good predictor of patient outcomes, and may provide valuable information for early intervention and treatment planning.

Furthermore, as alluded to by Lewis^46^, insight cannot be measured entirely objectively like a biological measure which relies, to some degree, on patient accounts. However, the vast majority of patients with psychotic disorders deny having a mental illness^1,2^ from first presentation,^48^ suggesting that insight and psychopathology are semi-independent domains.^49^ Nonetheless, no technology, including artificial intelligence, enables clinicians to read the thoughts of patients, thus leaving insight assessment based solely on traditional face-to-face approaches such as interviews and questionnaires, which may increase the likelihood of a patient adapting their answers to satisfy the clinician or researcher. Responses may be indicative of conflicting motivational dynamics: individuals may deny an illness they recognise as a means of preserving self-image or, alternatively, express acceptance of an illness they do not genuinely endorse, potentially to satisfy perceived expectations from clinicians or to increase the likelihood of hospital discharge. Some degree of expertise from the interviewer is therefore needed. While self-report measures of insight may reduce any censorship, they may provide an overly simplistic measure of insight. Also, previous results for the agreement between researcher-rated and self-report measures are mixed.^50^ Of relevance, the present SUMD-based proxy measure for assessment of insight in psychosis may have overcome these issues. In particular, it is worth noting that no cooperation from patients – this validation study was based on EHR – or significant rater experience were required, while the proxy SUMD has been shown to correlate strongly with the three insight dimensions from SUMD, namely awareness of having a mental illness, awareness of the need for treatment and awareness of the social consequences of the disorder.

### Methodological limitations

Three potential limitations should be considered when interpreting the study findings. First, this proxy instrument provided an unidimensional, three-point Likert score to measure such a complex phenomenological construct as insight in psychosis, which may therefore fail to capture its multidimensionality. It is important to note, however, that this unidimensional proxy measure of insight has been shown to have a robust correlation with the three principal insight domains of SUMD. Second, this study relied on previously collected data from the PAFIP programme, and only the three main SUMD items were considered, which may have affected content validity. More specifically, potential correlations of insight levels with severity of specific symptoms, such as hallucinatory experiences, delusions, disorganised thoughts, blunted affect, anhedonia or lack of sociability, could not be assessed. This is a common limitation of studies using SUMD, given the great heterogeneity in its administration.^41^ Third, the proxy tool was validated using EHR from a FEP cohort receiving care under an EIS, which tend to be more resourced than non-EIS mental health settings,^51^ including higher-quality documentation, which may have limited the generalisability of our results. Finally, inclusion of an external validation cohort was not feasible, which should be acknowledged as an important limitation of the present study.

### Clinical implications and future directions for research

The validation of this proxy version for SUMD has clinical implications. Since the original SUMD has been primarily employed in clinical trials and epidemiological studies, and to a lesser extent in clinical practice, this proxy version may aid in generating insight data from real-world patients, thus making a major contribution to practice-oriented research. This proxy measure may also be useful in standardising clinical data from case report-based systematic reviews, thereby facilitating research into less common psychotic disorders such as shared delusional disorder or Huntington’s disease schizophrenia-like psychosis. Also, this EHR-based proxy SUMD may have implications for management – for instance, as an outcome measure when auditing and monitoring mental health services in the psychosis pathway. The observed correlations between the proxy and other validated psychometric instruments, such as BPRS, SAPS and SANS, replicated the well-known relationship between lack of insight and psychotic symptom severity – poorer insight and worse psychosis.^52^ However, future studies should investigate whether this proxy for insight can also predict other outcomes such as psychotic relapse,^53^ suicidal behaviour^15^ and/or quality of life.^18^ Whether the SUMD proxy can be administered by other mental health professionals, such as nurses or social workers, remains to be demonstrated. In addition, it would be interesting to replicate these findings in less resourced mental health services – for instance, in low- and middle-income countries. Finally, this proxy may be automated in EHR-based research applying natural language-processing techniques,^54^ which requires further research.

In conclusion, while the assessment of clinical insight is paramount in the management of patients with psychotic disorders, previous validated psychometric scales remain underutilised in daily practice, representing an unmet clinical need. Alternatively, the proposed SUMD-based proxy tool for assessment of insight using EHR notes may offer a feasible, valid and reliable alternative to standard psychometric procedures for measuring insight in real-world settings. This proxy SUMD may be a particularly valuable tool in patient-oriented research, thus bridging the gap between research and routine clinical care in mental health.

## Data Availability

The SUMD proxy measure, which was developed and used in this study, is available to download from the supplementary material. Any additional statistical data that support the findings of this article are available from the corresponding author upon reasonable request.

## References

[1] Carpenter WT , Strauss JS , Bartko JJ. Flexible system for the diagnosis of schizophrenia: report from the WHO International Pilot Study of Schizophrenia. Science 1973; 182: 1275–8. 4752222 10.1126/science.182.4118.1275

[2] Amador XF , Flaum M , Andreasen NC , Strauss DH , Yale SA , Clark SC , et al. Awareness of illness in schizophrenia and schizoaffective and mood disorders. Arch Gen Psychiatry 1994; 51: 826–36. 7944872 10.1001/archpsyc.1994.03950100074007

[3] Ayesa-Arriola R , Rodríguez-Sánchez JM , Morelli C , Pelayo-Terán JM , Pérez-Iglesias R , Mata I , et al. Insight dimensions in first-episode psychosis patients: clinical, cognitive, pre-morbid and socio-demographic correlates. Early Interv Psychiatry 2011; 5: 140–9. 21352512 10.1111/j.1751-7893.2010.00249.x

[4] Cuesta MJ , Peralta V , Campos MS , Garcia-Jalon E. Can insight be predicted in first-episode psychosis patients? A longitudinal and hierarchical analysis of predictors in a drug-naive sample. Schizophr Res 2011; 130: 148–56. 21632216 10.1016/j.schres.2011.04.032

[5] Campos MS , Garcia-Jalon E , Gilleen JK , David AS , Peralta VM , Cuesta MJ. Premorbid personality and insight in first-episode psychosis. Schizophr Bull 2011; 37: 52–60. 20974749 10.1093/schbul/sbq119 PMC3004187

[6] Pousa E , Brebion G , Lopez-Carrilero R , Ruiz AI , Grasa E , Barajas A , et al. Predictors of clinical insight in first-episode psychosis: different patterns in men and women. Psychiatry Res 2024; 339: 116036. 38964140 10.1016/j.psychres.2024.116036

[7] Amador XF , Strauss DH , Yale SA , Gorman JM. Awareness of illness in schizophrenia. Schizophren Bull 1991; 17: 113–32. 10.1093/schbul/17.1.113 2047782

[8] David AS. Insight and psychosis. Br J Psychiatry 1990; 156: 798–808. 2207510 10.1192/bjp.156.6.798

[9] David AS. Insight and psychosis: the next 30 years. Br J Psychiatry 2020; 217: 521–3. 31685039 10.1192/bjp.2019.217

[10] Subotnik KL , Ventura J , Hellemann GS , Zito MF , Agee ER , Nuechterlein KH. Relationship of poor insight to neurocognition, social cognition, and psychiatric symptoms in schizophrenia: a meta-analysis. Schizophr Res 2020; 220: 164–71. 32334936 10.1016/j.schres.2020.03.038

[11] Canal-Rivero M , Ayesa-Arriola R , Ruiz-Veguilla M , Ortiz-García de la Foz V , Labad J , Crespo-Facorro B. Insight trajectories and their impact on psychosocial functioning: a 10-year follow-up study in first episode psychosis patients. J Psychopathol Clin Sci 2022; 131: 808–16. 36222628 10.1037/abn0000776

[12] Lysaker PH , Pattison ML , Leonhardt BL , Phelps S , Vohs JL. Insight in schizophrenia spectrum disorders: relationship with behavior, mood and perceived quality of life, underlying causes and emerging treatments. World Psychiatry 2018; 17: 12–23. 29352540 10.1002/wps.20508 PMC5775127

[13] Belvederi Murri M , Respino M , Innamorati M , Cervetti A , Calcagno P , Pompili M , et al. Is good insight associated with depression among patients with schizophrenia? Systematic review and meta-analysis. Schizophr Res 2015; 162: 234–47. 25631453 10.1016/j.schres.2015.01.003

[14] Ayesa-Arriola R , Alcaraz EG , Hernández BV , Pérez-Iglesias R , López Moríñigo JD , Duta R , et al. Suicidal behaviour in first-episode non-affective psychosis: specific risk periods and stage-related factors. Eur Neuropsychopharmacol 2015; 25: 2278–88. 26475577 10.1016/j.euroneuro.2015.09.008

[15] Ayesa-Arriola R , Terán JMP , Moríñigo JDL , Rivero MC , Setién-Suero E , Al-Halabi S , et al. The dynamic relationship between insight and suicidal behavior in first episode psychosis patients over 3-year follow-up. Eur Neuropsychopharmacol 2018; 28: 1161–72. 30097249 10.1016/j.euroneuro.2018.05.005

[16] López-Moríñigo JD , Ramos-Ríos R , David AS , Dutta R. Insight in schizophrenia and risk of suicide: a systematic update. Compr Psychiatry 2012; 53: 313–22. 21821236 10.1016/j.comppsych.2011.05.015

[17] Lopez-Morinigo J-D , Di Forti M , Ajnakina O , Wiffen BD , Morgan K , Doody GA , et al. Insight and risk of suicidal behaviour in two first-episode psychosis cohorts: effects of previous suicide attempts and depression. Schizophr Res 2019; 204: 80–9. 30253893 10.1016/j.schres.2018.09.016

[18] Davis BJ , Lysaker PH , Salyers MP , Minor KS. The insight paradox in schizophrenia: a meta-analysis of the relationship between clinical insight and quality of life. Schizophr Res 2020; 223: 9–17. 32763114 10.1016/j.schres.2020.07.017

[19] Lysaker PH , Roe D , Yanos PT. Toward understanding the insight paradox: internalized stigma moderates the association between insight and social functioning, hope, and self-esteem among people with schizophrenia spectrum disorders. Schizophr Bull 2007; 33: 192–9. 16894025 10.1093/schbul/sbl016 PMC2012366

[20] Lopez-Morinigo JD , David AS. Is too much insight bad for you? Br J Psychiatry 2024; 225: 454–7. 39422143 10.1192/bjp.2024.173

[21] Mervis JE , Vohs JL , Lysaker PH. An update on clinical insight, cognitive insight, and introspective accuracy in schizophrenia-spectrum disorders: symptoms, cognition, and treatment. Expert Rev Neurother 2022; 22: 245–55. 35244496 10.1080/14737175.2022.2049757

[22] Wing JK , Cooper JE , Sartorius N , Wing JK , Cooper JE , Sartorius N. Measurement and Classification of Psychiatric Symptoms: an Instruction Manual for the PSE and Catego Programm 1st ed. Cambridge University Press, 1974.

[23] McEvoy JP , Apperson LJ , Appelbaum PS , Ortlip P , Brecosky J , Hammill K , et al. Insight in schizophrenia. Its relationship to acute psychopathology. J Nerv Ment Dis 1989; 177: 43–7. 2562850 10.1097/00005053-198901000-00007

[24] Amador XF , Strauss DH , Yale SA , Flaum MM , Endicott J , Gorman JM. Assessment of insight in psychosis. Am J Psychiatry 1993; 150: 873–9. 8494061 10.1176/ajp.150.6.873

[25] Kemp R , David AS. Insight and Compliance. Treatment Compliance and the Therapeutic Alliance. Harwood Academic Publishers, 1997.

[26] Birchwood M , Smith J , Drury V , Healy J , Macmillan F , Slade M. A self-report Insight Scale for psychosis: reliability, validity and sensitivity to change. Acta Psychiatr Scand 1994; 89: 62–7. 7908156 10.1111/j.1600-0447.1994.tb01487.x

[27] Marková IS , Berrios GE. The assessment of insight in clinical psychiatry: a new scale. Acta Psychiatr Scand 1992; 86: 159–64. 1529740 10.1111/j.1600-0447.1992.tb03245.x

[28] Kay SR , Fiszbein A , Opler LA. The positive and negative syndrome scale (PANSS) for schizophrenia. Schizophr Bull 1987; 13: 261–76. 3616518 10.1093/schbul/13.2.261

[29] Guy W , Ban TA. The AMDP-System: Manual for the Assessment and Documentation of Psychopathology. Springer-Verlag, 1982.

[30] Østergaard SD , Opler MGA , Correll CU. Bridging the measurement gap between research and clinical care in schizophrenia: positive and negative syndrome scale-6 (PANSS-6) and other assessments based on the Simplified Negative and Positive Symptoms Interview (SNAPSI). Innov Clin Neurosci 2017; 14: 68–72. 29410939 PMC5788253

[31] Lopez-Morinigo J-D , Martínez AS-E , Barrigón ML , Escobedo-Aedo P-J , Ruiz-Ruano VG , Sánchez-Alonso S , et al. A pilot 1-year follow-up randomised controlled trial comparing metacognitive training to psychoeducation in schizophrenia: effects on insight. Schizophrenia (Heidelb) 2023; 9: 7. 36717598 10.1038/s41537-022-00316-x PMC9886217

[32] Hrisos S , Eccles MP , Francis JJ , Dickinson HO , Kaner EFS , Beyer F , et al. Are there valid proxy measures of clinical behaviour? A systematic review. Implement Sci 2009; 4: 37. 19575790 10.1186/1748-5908-4-37 PMC2713194

[33] Sanz-Gomez S , Alacreu-Crespo A , Guija JA , Giner L. Reliability and validity of proxy reports of impulsivity and aggression: An evidence-based assessment approach to psychological autopsy methods. Span J Psychiatry Ment Health 2023; 18: 28–33. 37979784 10.1016/j.sjpmh.2023.10.003

[34] Kirkpatrick B , Buchanan RW , Breier A , Carpenter WT. Case identification and stability of the deficit syndrome of schizophrenia. Psychiatry Res 1993; 47: 47–56. 8516416 10.1016/0165-1781(93)90054-k

[35] López-Díaz Á. , Ayesa-Arriola R , Garrido-Torres N , Otíz-García de la Foz V , Suárez-Pinilla P , Ramírez-Bonilla ML , et al. A proxy measure of premorbid adjustment in psychosis for large-scale epidemiological studies and electronic health record-based research. Schizophr Res 2022; 243: 467–9. 35288002 10.1016/j.schres.2022.02.034

[36] López-Díaz Á. , Palermo-Zeballos FJ , Gutierrez-Rojas L , Alameda L , Gotor-Sánchez-Luengo F , Garrido-Torres N , et al. Proxy measures for the assessment of psychotic and affective symptoms in studies using electronic health records. BJPsych Open 2024; 10: e22. 38179604 10.1192/bjo.2023.623 PMC10790217

[37] Lopez-Diaz A , Ayesa-Arriola R , Ortiz-Garcia de la Foz V , Suarez-Pinilla P , Ramirez-Bonilla ML , Vazquez-Bourgon J , et al. Predictors of diagnostic stability in brief psychotic disorders: findings from a 3-year longitudinal study. Acta Psychiatr Scand 2021; 144: 578–88. 34431080 10.1111/acps.13364

[38] Pelayo-Teran JM , Perez-Iglesias R , Ramirez-Bonilla M , Gonzalez-Blanch C , Martinez-Garcia O , Pardo-Garcia G , et al. Epidemiological factors associated with treated incidence of first-episode non-affective psychosis in Cantabria: insights from the Clinical Programme on Early Phases of Psychosis. Early Interv Psychiatry 2008; 2: 178–87. 21352151 10.1111/j.1751-7893.2008.00074.x

[39] von Elm E , Altman DG , Egger M , Pocock SJ , Gotzsche PC , Vandenbroucke JP , et al. The Strengthening the Reporting of Observational Studies in Epidemiology (STROBE) statement: guidelines for reporting observational studies. Lancet 2007; 370: 1453–7. 18064739 10.1016/S0140-6736(07)61602-X

[40] Andreasen NC , Carpenter WT , Kane JM , Lasser RA , Marder SR , Weinberger DR. Remission in schizophrenia: proposed criteria and rationale for consensus. Am J Psychiatry 2005; 162: 441–9. 15741458 10.1176/appi.ajp.162.3.441

[41] Dumas R , Baumstarck K , Michel P , Lancon C , Auquier P , Boyer L. Systematic review reveals heterogeneity in the use of the Scale to Assess Unawareness of Mental Disorder (SUMD). Curr Psychiatry Rep 2013; 15: 361. 23636985 10.1007/s11920-013-0361-8

[42] Michel P , Baumstarck K , Auquier P , Amador X , Dumas R , Fernandez J , et al. Psychometric properties of the abbreviated version of the Scale to Assess Unawareness in Mental Disorder in schizophrenia. BMC Psychiatry 2013; 13: 229. 24053640 10.1186/1471-244X-13-229 PMC3851247

[43] Masand P , OGorman C , Mandel FS. Clinical Global Impression of Improvement (CGI-I) as a valid proxy measure for remission in schizophrenia: analyses of ziprasidone clinical study data. Schizophr Res 2011; 126: 174–83. 21185155 10.1016/j.schres.2010.10.024

[44] Brent BK , Giuliano AJ , Zimmet SV , Keshavan MS , Seidman LJ. Insight into illness in patients and caregivers during early psychosis: a pilot study. Schizophr Res 2011; 127: 100–6. 21315560 10.1016/j.schres.2010.12.024

[45] Grover S , Chakrabarti S , Ghormode D , Dutt A , Kate N , Kulhara P. Clinicians versus caregivers’ ratings of burden in patients with schizophrenia and bipolar disorder. Int J Soc Psychiatry 2014; 60: 330–6. 23788439 10.1177/0020764013488708

[46] Lewis A. The psychopathology of insight. Br J Med Psychol 1934; 14: 332–48.

[47] Sanz M , Constable G , Lopez-Ibor I , Kemp R , David AS. A comparative study of insight scales and their relationship to psychopathological and clinical variables. Psychol Med 1998; 28: 437–46. 9572100 10.1017/s0033291797006296

[48] Ayesa-Arriola R , Moríñigo JDL , David AS , Pérez-Iglesias R , Rodríguez-Sánchez JM , Crespo-Facorro B. Lack of insight 3 years after first-episode psychosis: an unchangeable illness trait determined from first presentation? Schizophr Res 2014; 157: 271–7. 24934905 10.1016/j.schres.2014.05.011

[49] Cuesta MJ , Peralta V , Zarzuela A. Reappraising insight in psychosis. Multi-scale longitudinal study. Br J Psychiatry 2000; 177: 233–40. 11040884 10.1192/bjp.177.3.233

[50] Capdevielle D , Norton J , Aouizerate B , Berna F , Chereau I , DAmato T , et al. Comparison of three scales (BIS, SUMD and BCIS) for measuring insight dimensions and their evolution after one-year of follow-up: findings from the FACE-SZ cohort. Psychiatry Res 2021; 303: 114044. 34161854 10.1016/j.psychres.2021.114044

[51] OConnell N , O’Connor K , McGrath D , Vagge L , Mockler D , Jennings R , et al. Early Intervention in Psychosis services: a systematic review and narrative synthesis of the barriers and facilitators to implementation. Eur Psychiatry 2021; 65: e2. 34913421 10.1192/j.eurpsy.2021.2260 PMC8792869

[52] Mintz AR , Dobson KS , Romney DM. Insight in schizophrenia: a meta-analysis. Schizophr Res 2003; 61: 75–88. 12648738 10.1016/s0920-9964(02)00316-x

[53] Berge D , Mane A , Salgado P , Cortizo R , Garnier C , Gomez L , et al. Predictors of relapse and functioning in first-episode psychosis: a two-year follow-up study. Psychiatr Serv 2016; 67: 227–33. 26467909 10.1176/appi.ps.201400316

[54] Jackson RG , Patel R , Jayatilleke N , Kolliakou A , Ball M , Gorrell G , et al. Natural language processing to extract symptoms of severe mental illness from clinical text: the Clinical Record Interactive Search Comprehensive Data Extraction (CRIS-CODE) project. BMJ Open 2017; 7: e012012. 10.1136/bmjopen-2016-012012 PMC5253558 28096249

